# Ten simple rules to increase computational skills among biologists with Code Clubs

**DOI:** 10.1371/journal.pcbi.1008119

**Published:** 2020-08-27

**Authors:** Ada K. Hagan, Nicholas A. Lesniak, Marcy J. Balunas, Lucas Bishop, William L. Close, Matthew D. Doherty, Amanda G. Elmore, Kaitlin J. Flynn, Geoffrey D. Hannigan, Charlie C. Koumpouras, Matthew L. Jenior, Ariangela J. Kozik, Kathryn McBride, Samara B. Rifkin, Joshua M. A. Stough, Kelly L. Sovacool, Marc A. Sze, Sarah Tomkovich, Begum D. Topcuoglu, Patrick D. Schloss

**Affiliations:** 1 Department of Microbiology and Immunology, University of Michigan, Ann Arbor, Michigan, United States of America; 2 Division of Medicinal Chemistry, Department of Pharmaceutical Sciences, University of Connecticut, Storrs, Connecticut, United States of America; 3 Department of Internal Medicine, University of Michigan, Ann Arbor, Michigan, United States of America; 4 Department of Computational Medicine and Bioinformatics, University of Michigan, Ann Arbor, Michigan, United States of America; Carnegie Mellon University, UNITED STATES

## Introduction

For most biologists, the ability to generate data has outpaced the ability to analyze those data. High throughput data comes to us from DNA and RNA sequencing, flow cytometry, metabolomics, molecular screens, and more. Although some accept the approach of compartmentalizing data generation and data analysis, we have found scientists feel empowered when they can both ask and answer their own biological questions [1]. In our experience performing microbiome research, it is more common to find exceptional bench scientists who are inexperienced at analyzing large data sets than to find the reverse. Of course, this raises a challenge: how do we train bench scientists to effectively answer biological questions with these larger data sets?

The standard undergraduate and graduate training in the biological sciences is typically insufficient for developing these skills [2]. To fill this void, there has been growth in online data science training resources through companies including Data Camp (https://www.datacamp.com) and Codecademy (https://www.codecademy.com), massive open online courses (MOOCs), and in-person workshops hosted by organizations, including The Carpentries (https://carpentries.org). Each of these formats are highly popular among their audience (e.g., [3]); however, recent analyses of workshops suggest that they have little impact on the long-term retention of the material that is taught [4]. This result should not be surprising. These learning formats ask participants to engage in massed practice, an approach that is known to be ineffective [5–8]. In contrast, participants need regular opportunities to engage in repeated deliberate practice that encourage them to practice their retrieval and application of the material [9,10]. There is clearly a need to develop a framework that can build upon material introduced in coursework and workshops and that can help researchers stay up to date on the latest best practices, algorithms, and tools.

To address this need, we noted the similarity between the feeling of unease in analyzing the data and the struggles scientists face with engaging the voluminous scientific literature. A common strategy for keeping up with the literature is to participate in a Journal Club. Regardless of the discipline, a Journal Club involves group discussion of a preselected paper that can range from informal discussions to PowerPoint presentations and course credit. In addition to building upon material from traditional coursework and staying current on the literature, Journal Clubs help strengthen skills in critical thinking, communication, and integrating the literature [11]. Because most Journal Clubs occur on a regular schedule, they are effective by virtue of repeated practice. With this model in mind, over the past four years we have experimented with creating a Code Club model with the goal of improving reproducible data analysis skills in a laboratory environment.

Our Code Club sessions are an hour long and alternate with our lab’s Journal Club as the second part of weekly two-hour lab meetings. Initially, the Code Club was used to review code from trainee projects. Instead of a presentation, the presenter would project their code onto a screen and the participants would go through the code, stating the logic behind each line. This approach emphasized the importance of code readability and gave beginners the opportunity to see the real-life, messy code of more experienced peers. Unfortunately, the format only allowed us to review a fraction of a project’s code, making it difficult to integrate the programmer’s logic across their full project. A major issue with this model was that sometimes beginners could not contribute to improving the code, and even when they could, more experienced group members would eventually dominate the discussion. This led to a lack of participation by beginners, who would sometimes mentally withdraw, resulting in an adversarial environment between the presenter and more experienced members. As a result, presenters were reluctant to offer to present again.

From these experiences, we began a collective conversation to improve our Code Club model. We have identified two successful approaches. The first is a more constructive version of a group code review. The presenter clearly states the problem they want to solve, breaks the participants into smaller groups, and then asks each group to solve the problem, or a portion of it. For example, someone may have an R script with a repeating chunk of code. The challenge for the session would be to convert the code chunk into a function to be called throughout the script to make it “DRY” (i.e., Don’t Repeat Yourself [12]). The presenter leaves the session with several partial or working solutions to their problem, and the importance of writing DRY code is reinforced. The second approach is a tutorial. The presenter introduces a new package or technique and assigns an activity to practice the new approach. For example, at one Code Club, participants were given raw data and a finished plot. Paired participants were tasked with generating the plot from the data using R syntax from either the base language or the ggplot2 package. For this exercise, base R users had to use ggplot2 and vice versa. In either approach, the Code Club ends with a report back to the larger group describing the approach each pair took. We generally find that preparing a Code Club session takes similar effort to preparing a Journal Club presentation. Our Code Clubs typically have 7 to 10 participants, but the inherent “think-pair-share” approach should allow it to be scaled to groups of variable sizes [13].

We continue to experiment with approaches for running Code Club. For example, during the COVID-19 pandemic in 2020, when many research labs were shuttered, author PDS started posting Code Club materials (https://www.riffomonas.org/code_club/) that individuals or research groups can use to practice their coding skills. During this time, we continued our group’s Code Club sessions using the breakout room and screen sharing features in Zoom. Regardless of our approach to leading a Code Club, we have learned that it is critical for the presenter to clearly articulate their goals and facilitate participant engagement. Although some Code Club sessions may be more experimental than others, on the whole they are a critical tool to train bench scientists in reproducible data analysis practices. We have provided some examples of successful topics in Table 1. We have summarized the results of our own experimentation as Code Club presenters and participants into Ten Rules. The first 3 rules apply to all participants, Rules 4 through 8 are targeted to presenters, and the last two for non-presenters. As the rules describe, this model can easily be adapted to groups of non-biologists or those with stronger backgrounds in computer science.

**Table 1:** Examples of successful Code Club topics.

Title	Description
base versus ggplot2	Given input data and a figure, recreate the figure using R’s base graphics or ggplot2 syntax
Snakemake	Given a bash script that contains an analysis pipeline, convert it to a Snakemake workflow (https://github.com/SchlossLab/snakemake_riffomonas_tutorial)
DRYing code	Given script with repeated code, create functions to remove repetition
Vegan R package	Compare microbial communities using the adonis function in the Vegan R package (https://github.com/SchlossLab/Code_Review_42717)
tidy data	Given a wide-formatted data table, convert it to a long, tidy-formatted data table using tools from R’s tidyverse
GitFlow	Participants file and claim an issue to add their name to a README file in a GitHub-hosted repository and file a pull request to complete the issue
googledocs4 R package	Scrape a Google docs workbook and clean the data to identify previous Code Club presenters
Develop an R Package	Convert a lab member’s collection of scripts into an R package over a series of sessions
Documenting R code	Use roxygen2 to supplement comments in R code to improve documentation (https://github.com/SchlossLab/documenting-R)
gganimate R package	Convert static plots generated with ggplot2 package into GIFs (https://github.com/SchlossLab/2020-04-12-CodeClub_PlotAnimation/)

## Rule 1: Reciprocate respect

It is critical that the presenter and participants respect each other and that a designated individual (e.g., the lab director) enforces a code of conduct. Each member of the Code Club must have the humility to acknowledge that they have more to learn about any given topic. We have found that many problems are avoided when the presenter takes charge of the session with a clear lesson plan, thoughtfully creates groups, and gives encouragement. Similarly, participants foster a positive environment by remembering that the task is not a competition, instead focusing on the presenter’s goals, allowing their partner to contribute, asking clarifying questions when appropriate, and avoiding distractions (e.g., email, social media). Learning to program is challenging. Too often it is attempted in an environment with nonconstructive criticism. All parties in a Code Club are responsible for preventing this by demonstrating respect for themselves and their colleagues.

## Rule 2: Let the material change you

Part of the humility required to participate in a Code Club is acknowledging that your training is incomplete and that it is possible for everyone to learn something new. For participants, assume that the presenter has a plan and follow their presented approach. After the Code Club, try to incorporate that material into new code or refactor old code. By practicing the material in a different context, you will learn the material better. For presenters, incorporate suggested changes into your code. Either party may identify concepts that they are unsure of, presenting opportunities for further conversation and learning.

## Rule 3: Experiment!

The selection of content and structure for each Code Club works best when it is democratic and distributed. If someone thinks a technique is worth learning and wants to teach it, they have that power. If they want to experiment with a different format, they are free to try it out. Members of the Code Club need to feel like they have the power to shape the direction of the group. If members are following these Rules, they will naturally reflect on the skills and interests of the other members in the group. For example, there is always turnover in a research group, making it important to revisit basic concepts to teach to new people and provide a refresher to others. One successful experiment found that we could keep basic content interesting to more experienced group members by creating related problems that varied in their difficulty. The group should also feel free to experiment with the format and incorporate group feedback by ending with a debrief discussing the pros and cons of new formats.

## Rule 4: Set specific goals

In our early Code Clubs, we noticed that if the presenter did not clearly state their goals for the session, it often led to frustration for both the presenter and participants. If the presenter shared their own code, did they want participants to focus on their coding style or did they want help incorporating a new package into their workflow? Participants will always notice or ask about code concepts that are not the focus of the exercise; a presenter with a specific goal can bring tangential conversations back to the planned task. Where possible, presenters should create a simplified scenario (i.e., minimal, reproducible example [14]), which can be helpful in focusing the participants. The presenter should verify ahead of time that the simplified example works and behaves the way they expect. Beyond the content, clear goals for participant activities will help both parties stay on task and avoid frustration. For more advanced learners, the presenter can create stretch goals or give an activity with multiple stopping points where participants would feel successful (e.g., commenting code, creating function, implementing function, refactoring function). Accomplishing specific goals is more likely to result in a positive outcome for presenters and participants.

## Rule 5: Keep it simple

Our Code Club needs to fit within an hour time slot. When considering their Code Club activity, the presenter should plan for an introduction and brief instruction, time for participants to engage the material, and time for everyone to report back within that hour. A typical schedule for Code Club is 10 minutes of introduction and instruction, 30 minutes of paired programming, 5 minutes to get groups to wrap up, and 10 minutes to report back to the group. We once had a presenter try to teach basic, but unfamiliar, Julia syntax. Unfortunately, the time was up before the participants had installed the interpreter. Some tips to help keep it simple are to limit the presented code chunks to less than 50 lines or, conversely, consider the number of lines that might be required to accomplish a solution. Remember that learners may need up to three times as long to complete a task that is straightforward for the presenter, so Code Club is best kept simple.

## Rule 6: Give participants time to prepare

Similar to a Journal Club, the presenter should give participants a few days (ideally a week) to prepare for the Code Club. Considering the compressed schedule described in Rule 5, asking participants to download materials beforehand is helpful to ensure a quick start. The presenter should provide the participants with instructions on how to install dependencies, download data, and get the initial code. This might also uncover weak points in the presenter’s plan and enable them to ensure that the materials work as intended before the Code Club. We have found that using GitHub repositories for each Code Club can help make information, scripts, and data easily available to participants. Although this can be convenient, introducing GitHub on top of the session’s activities can impose a significant cognitive load and frustration to those not already comfortable with GitHub. Perhaps a first Code Club could introduce using git and GitHub to engage in collaborative coding. A lower barrier entry point for the presenter is posting their code, data, and information in a lab meeting-dedicated Slack channel or via email. Whatever method is used, the presenter should be sure to communicate the topic and necessary materials with the participants ahead of time.

## Rule 7: Don’t give participants busywork

Participants want to learn topics that will either be useful to them or help their colleague (i.e., the presenter). Presenters should do their best to satisfy those motivations, whether it is through the relevancy of the concept or the data. It does not make sense to present a Code Club on downloading stock market data if it is not useful or interesting to the group. Similarly, participants should not be tasked with improving the presenter’s code if the presenter has no intention of incorporating the suggestions. A list of packages or tasks that group members are interested in along with a log of previous topics could help a presenter struggling to find a topic choose one that will make a rewarding Code Club.

## Rule 8: Include all levels of participants

As suggested by Rule 7, a significant challenge to presenting at Code Club is selecting topics and activities that appeal to a critical mass of the participants. This is particularly difficult if participants have a wide range of coding experience, which can follow a turnover in group membership. Beginners will benefit from sessions that cover fundamental concepts and functions. The benefits for more experienced participants include the improved understanding of concepts by teaching and breaking down problems into simpler elements. Furthermore, they can benefit by contributing to an environment of learning that they previously benefited from. In addition, instead of focusing on core functions, the group could balance Code Club sessions that cover basics with those that introduce new methods and packages to the group. We have also identified several strategies to overcome the challenges presented by participants at varying skill levels. Central to the Code Club format is the use of paired programming [15]. Instead of letting participants form their own pairs, the presenter can select pairs of participants with either similar or differing skill levels, depending on the presenter’s goals. Partnerships between those with similar skill levels require the presenter to design appropriate activities for each skill level. We have found that commenting code is a good skill for beginners because it forces them to dissect and understand a code chunk line by line. It also reinforces the value of commenting as they develop their skills and independence. An advantage of forming partnerships between people with disparate skill levels is that it is more likely for groups to provide the presenter with a diverse range of methods that achieve the same result. This approach to pairing also helps to graft new members that have emerging programming skills. Regardless of how partners are selected, consider asking the pairs to identify a navigator and a driver [16]. The driver types at the computer while the navigator tells them what to type, thus ensuring participation of both partners. Midway through the activity, the presenter can have the partners switch roles. Intentionally forming pairs can also engineer group interactions by avoiding potentially disruptive partnerships or pairing reliable role models with new group members.

## Rule 9: Prepare in advance to maximize participation

It is not possible to fully participate in a Journal Club discussion about a paper that the participant has not read. In that context, coming to Code Club without having installed a dependency is similar to asking a simple question about the Journal Club paper without first reading it. Both instances show a lack of preparation. Just as a presenter must follow Rule 6 to provide materials ahead of time, participants must review the code in advance, download the data sets, install the necessary packages, and perhaps read up on the topic. If the Code Club is based on a paper or chapters in a data science book (e.g., [17–19]), the participants should read them before the session and consider how they might incorporate the concepts into their own work.

## Rule 10: Participate

An essential ingredient of any Code Club is active participation from all parties. Having an open laptop on the table and permission to use it can feel like an invitation to get distracted by other work, emails, and browsing the internet; fight that urge and focus on the presenter’s goals. Be respectful and allow your partner to contribute. Speak up for yourself and force your partner to let you contribute. If the material seems too advanced for you, it can be frustrating, and tempting to mentally check out. Fortunately, programming languages like R and Python are generally expressive, which should allow you to engage with the logic, even if the syntax is too advanced. Oftentimes, understanding the logic of when to use one modeling approach over another is more important than knowing how to use it. If you understand the “why,” the “how” will quickly follow. More experienced participants should aim to communicate feedback and coding suggestions at a level that all participants can understand and engage in. Regardless of skill levels, your partner and the presenter put themselves in a vulnerable position by revealing what they do or do not know. Encourage them and show your gratitude for helping you learn something new by fully participating in each Code Club.

## Conclusion

The most important rules are the first and last. Members of the Code Club need to feel comfortable with other group members and sufficiently empowered to try something new or ask for help. Aside from expanding our programming skills, we have noticed two other benefits that help create a positive culture. First, we have intentionally interviewed postdoctoral candidates on the days we hold Code Club. We make it clear that they are not being assessed on their coding skills, but instead use it as an opportunity to see how the candidate interacts with other members of the research group. At the same time, the candidate can learn about the culture of the research group through active participation. Second, members of other research groups have integrated themselves into our Code Club to minimize the isolation they feel in growing their skills within smaller research groups. This speaks to both the broader need for Code Club and the likelihood of success when expanded to include a larger group of individuals with broader research interests. There is no reason that the Code Club format would not work with groups independent of a research group as long as everyone follows the Rules. Finding data sets and applications that are interesting to a critical mass of people is essential to starting and sustaining such a group. Ultimately, Code Club has improved the overall data analysis skills, community, and research success of our lab by empowering researchers to seek help from their colleagues.
